# FANTOM: Functional and taxonomic analysis of metagenomes

**DOI:** 10.1186/1471-2105-14-38

**Published:** 2013-02-01

**Authors:** Kemal Sanli, Fredrik H Karlsson, Intawat Nookaew, Jens Nielsen

**Affiliations:** 1 Department of Chemical and Biological Engineering, Chalmers University of Technology, Kemivägen 10, Gothenburg, SE 412 96, Sweden; 2 Present Address: Department of Biological and Environmental Sciences, University of Gothenburg, Box 100, Gothenburg, S-405 30, Sweden

**Keywords:** Graphical User Interface (GUI), Metagenomics, Statistical analysis, Multivariate analysis, Visualization

## Abstract

**Background:**

Interpretation of quantitative metagenomics data is important for our understanding of ecosystem functioning and assessing differences between various environmental samples. There is a need for an easy to use tool to explore the often complex metagenomics data in taxonomic and functional context.

**Results:**

Here we introduce FANTOM, a tool that allows for exploratory and comparative analysis of metagenomics abundance data integrated with metadata information and biological databases. Importantly, FANTOM can make use of any hierarchical database and it comes supplied with NCBI taxonomic hierarchies as well as KEGG Orthology, COG, PFAM and TIGRFAM databases.

**Conclusions:**

The software is implemented in Python, is platform independent, and is available at http://www.sysbio.se/Fantom.

## Background

Metagenomics [1] is the culture independent study of an environmental sample by sequencing of the recovered genetic materials of targeted ribosomal RNAs (16S) through amplicon sequencing or whole genomic DNA. This allows for determining the ecosystems taxonomic diversity, functional capacity, dynamics and comparison with other environments. Typically for whole genome based metagenomics, extracted DNA from an environmental sample is a starting material to generate short reads of DNA through next generation sequencing (NGS) technologies that represent the microbiota of the sample. The generated raw sequence reads data typically contain errors that need to be eliminated before further steps using trimming and filtering processes based on a base calling quality score (Phred) [2,3]. The high quality reads can be annotated to reference taxonomic and functional features using sequence similarity based alignment methods i.e. BLAST [4], HMMER [5], etc. against reference databases. Another approach is based on mapping high quality reads on reference genomes or well annotated genes by short read aligners [6]. There are web services such as CAMERA [7], IMG/M [8] and MG-RAST [9], available for performing the above mentioned pipeline of NGS processing and annotation in an automated fashion. Depending on user-given parameters such as percentage similarity or e-value thresholds, each of these individual software tools or web services are able to report the annotated sequences in terms of abundance data for each feature in the subjected database. Further analysis of the hereby obtained quantitative abundance data of metagenomics features, in particular together with sample meta data is important for biological interpretation [10,11].

Although, the above mentioned web-services can to some extent provide both analysis tools for the comparative analysis of metagenomes, these methods have some limitations; 1) statistical and visual analysis capabilities are limited, 2) functional annotation sources might not satisfy user’s demand, and 3) users may simply not want to upload their sequencing data to an online service. There are several standalone software tools available for statistical analysis and visualization of annotated metagenomics data, e.g. MEGAN [12], SmashCommunity [13], STAMP [14], shotgunFunctionalizeR [15], VEGAN [16], QIIME [17] and Mothur [18].

We identified the requirement for a user-friendly comparative analysis and data visualization tool where annotated metagenomics data can meet sample metadata and be analyzed at different hierarchy levels using a built-in or user provided biological database. This tool, FANTOM for **F**unctional **AN**notation and **T**axonomic analysis **O**f **M**etagenomes, is an easy installed, standalone software tool that is accessed through a graphical user interface to analyze abundance of metagenomics features that are easily integrated with NCBI taxonomy, KEGG [19], COG [20] and protein family databases PFAM [21] and TIGRFAM [22] with hierarchy information. We believe that this tool will be highly useful for a broad community of scientists desiring to analyze metagenomics data.

## Implementation

The software installer, user manual and demonstration videos can be found and downloaded at the website http://www.sysbio.se/Fantom

FANTOM was implemented in Python allowing it to operate platform independent in addition to the utilization of core scientific packages including numpy, scipy and matplotlib to implement statistical functions and various plotting options. wxPython was incorporated to provide graphical user interface components and storm package was used for object relational mapping of data from the local SQLite database. The software was tested successfully on Windows, Linux and OSX operating systems and the installers are provided for the different platforms.

FANTOM requires two input files; a metagenomics abundance file, which could be derived from annotation of metagenomics data, including either taxonomic or functional annotations and another file containing the samples’ metadata (see user manual and demonstration videos). Besides, there are web services such as CAMERA [7], IMG/M [8] and MG-RAST [9] that allow the users to easily obtain metagenomics abundance from their metagenome data. Metadata can either be numerical or categorical and the software will automatically recognize the format and display options for selecting and filtering samples. Functional hierarchy information was downloaded from KEGG Orthology, COG, PFAM and TIGRFAM databases and taxonomic lineage information was downloaded from the NCBI taxonomy database and constitute the standards feature databases in the software package. Moreover, FANTOM provides the option that allows the user to create and use a custom made hierarchical database. The custom database can be easily imported as a tabular input file to analyze the abundances of corresponding database levels.

In FANTOM, the abundance can be specified at different levels in hierarchical databases, which are called nodes (e.g. pathways or Genera), the abundance of a higher node in the hiearchy is calculated by summing the abundance of all member nodes further down in the hierarchy structure (e.g. orthologs or species). The abundance of nodes that are members of more than one higher level nodes are split equally between higher nodes.

The metadata file can include both categorical and numerical properties of each sample, which can then be used in FANTOM to filter and select sample groups of interest for comparative analysis. Numerical variables can further be used for correlation analysis with the annotated features. Taxonomic or functional feature abundances can be displayed and processed either as absolute counts or as normalized relative values. After selecting relevant subsets of metagenomics data, principal component analysis can be applied to reduce the dimensionality. Furthermore, hierarchical clustering, another multivariate analysis method is implemented to evaluate high dimensional metagenomics data by drawing dendograms for features and samples as well as a heatmap with 2-dimensional clustering, reflecting abundance values.

**Figure 1:**
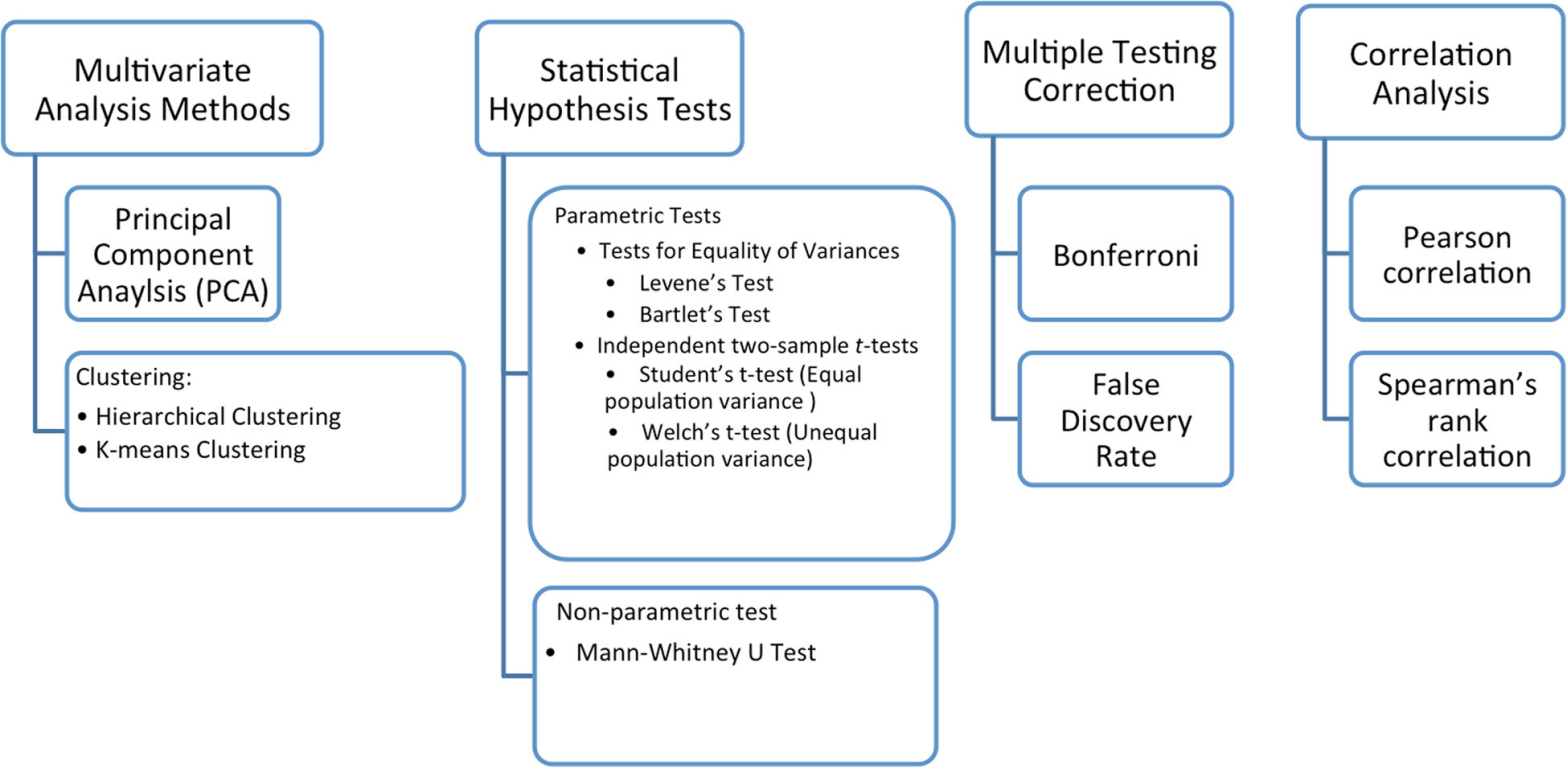
Statistical analyses provided in FANTOM.

By defining groups of samples based on metadata, statistical hypothesis tests can be performed to compare metagenomics features between groups. FANTOM, currently supports two sample comparisons. Non-parametric Mann-Whitney U test was implemented in FANTOM and is encouraged because of the typically non-normally distribution of metagenomics data. Shapiro Wilk’s normalty test, Bartlett’s test and Levene’s test for equality of variances and Student’s t-test were also implemented as parametric hypothesis tests. Obtained p-values of these tests can be adjusted for multiple testing using either Bonferroni or Benjamini-Hochberg false discovery rate (fdr). Results can finally be filtered according to p-values, absolute fold change and mean relative abundance. The multivariate and statistical methods that are provided in FANTOM are summarized in Figure 1.

**Figure 2:**
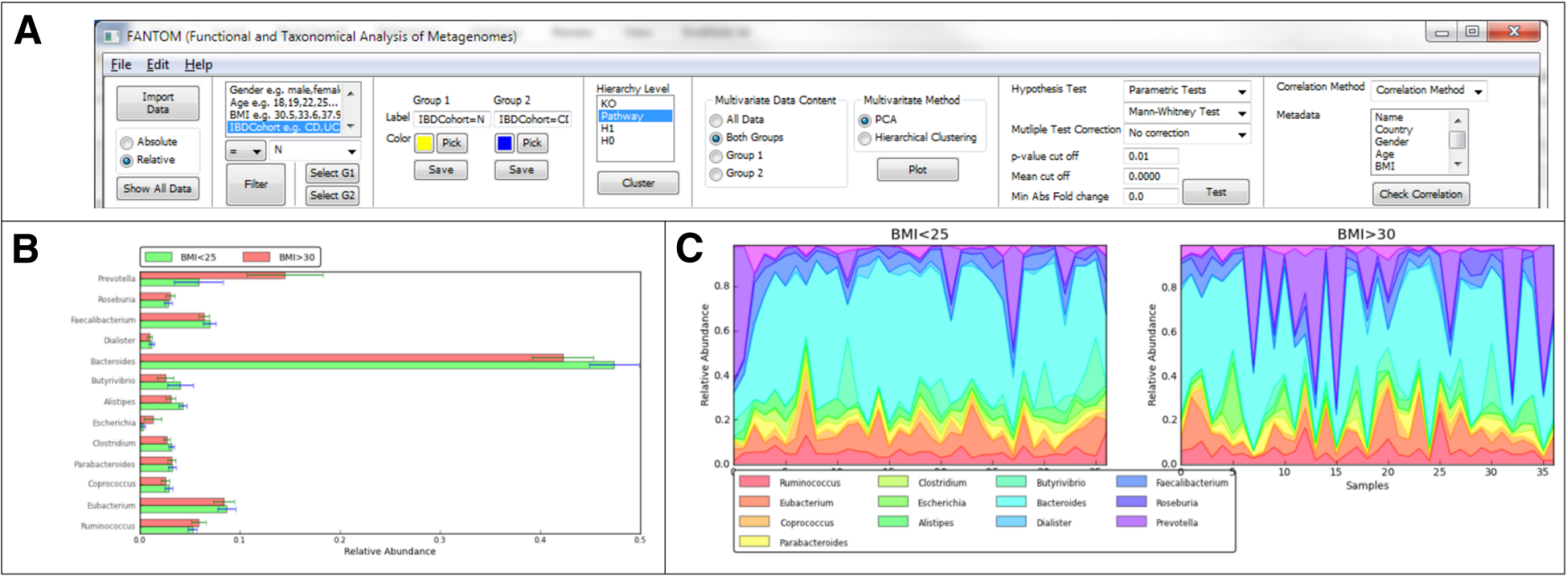
**Graphical user interface of FANTOM and examples of plots that can be generated. A**) FANTOM data manipulation panel **B**) Bar graph comparing two types of patients by KEGG pathway level abundances **C**) Area plot showing two sets of samples and individual profiles of KEGG pathway abundance in each sample.

FANTOM provides several options for graphical representation of the data and comparative analysis. After hypothesis testing, significant results can be displayed by bar charts, boxplots, pie charts and area plots. Plotting options make use of the hierarchies in NCBI taxanomy, KEGG and COG, groups of metagenomics data according to the specified level and added filtering options. The software provides means to save the figures in high quality formats that can be used directly for publication. An example of a screen shot of FANTOM is shown in Figure 2.

## Results and discussion

The software was evaluated using metagenomics data from the gut microbiome of 124 subjects in the MetaHIT [23] project. Sequences were quality trimmed (SolexaQA –p 0.05) and sequences shorter than 35 bp were filtered out. High quality reads were aligned to a reference catalogue of 440 genomes to obtain taxonomic abundance. Moreover, the reads were aligned to the MetaHIT gene catalogue of 3.3 million genes to get the abundance of genes. The genes were annotated to the KEGG and COG database and this information was used to transform gene abundance to KEGG KO and COG abundances. This data are available as example files together with metadata included bundled with the software.

The MetaHIT study focused on two human diseases, obesity and inflammatory bowel disease (Crohn’s disease and ulcerative colitis), which we make use of here as example capabilities of FANTOM.

**Figure 3:**
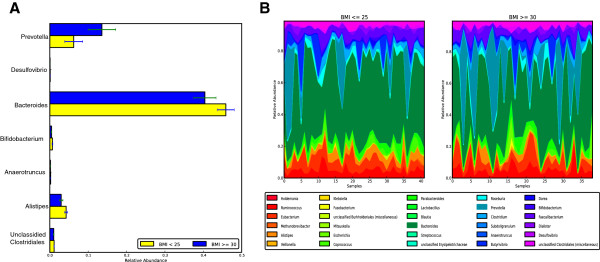
**Comparison of healthy lean subject with obese subjects. A**) Genera with a FDR < 0.2 that are differentially abundant between lean an obese subjects. **B**) Area plot of the significant species in **A**).

Differences based on Mann-Whitney U test (FDR < 0.2) were observed for lean (BMI < 25) and obese (BMI > 30) individuals in species and genus level taxonomy terms. At the genus level, particularily *Prevotella* was enriched in obese individuals whereas *Bacteroides*, *Bifidobacterium*, *Alistipes* and *unclassified Clostridiales* were enriched in normal weight subjects (Figure 3A). Previous reports have discussed the association between the ratio of Firmicutes to Bacteroidetes with obesity and came to different conclusions [24-26]. Here we observed changes within the Bacteroidetes phyla by an increase of *Prevotella* and a decrease in *Bacteroides* in obese subjects. To get an appreciation of the variability and profiles in the microbiota across individuals, the relative abundance profiles were plotted in area plots (Figure 3B).

**Figure 4:**
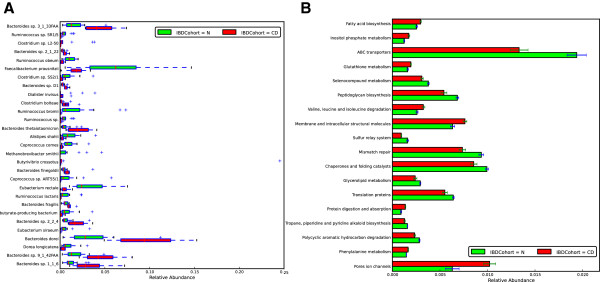
**Taxonomic and functional differences in Crohn’s disease (CD) patients compared to healthy subjects. A**) Differentially abundant species between CD and healthy subjects. **B**) Differentially abundant KEGG pathways between CD and healthy subjects.

Comparisons between Spanish Crohn’s disease (CD) patients and healthy individuals in taxonomical terms are illustrated in Figure 4A. Based on Mann-Whitney U test (p-value < 0.05), it is clearly seen that there was a decrease in CD patients of several common Firmicutes species commonly known to be present in a healthy gut such as *Ruminococcus sp, Faecalibacterium sp., Clostridium sp., Alistripes sp., Coprocouccus sp., Methanobrevibacter sp., Eubacterium sp. Dorea sp. and butyrate producing bacteria.* The loss of Firmicutes and *Faecalibacterium prausnitzii* in particular has been observed previously [27] and is confirmed here. Subsequently, an increase of several Bacteroides *sp.* was observed in CD patients. By using the functional information and testing for differential abundance of KEGG pathways between CD patients and healthy subjects specific metabolic pathways could be identified as seen in Figure 4B. The results are consistent with the taxonomical changes as the enrichment of the Gram negative *Bacteroides* sp. are consistent with the decreased number of genes for peptidoglycan biosynthesis as well as ABC transporter but an increase in membrane structure and transport as well as ion channels in CD patients.

## Conclusion

We provide an open source standalone user-friendly software tool, FANTOM, for data analyses and data mining of read counts from whole shotgun metagenomics or amplicon sequencing studies. FANTOM allows the user to integrate sample metadata, taxonomy and gene functional profiling in the analysis, and FANTOM is supplied with access to biological databases as well as the possibility to upload custom made databases.

## Availability and requirements

**Project name:** FANTOM : Functional and taxonomic analysis of metagenomes

**Project home page:**http://www.sysbio.se/Fantom

**Operating system(s):** Windows, Linux, Mac OSX

**Programming language:** python

**Other requirements:** -

**License:** GNU-GPL version 3 software license

**Any restrictions to use by non-academics:** No

## Competing interests

The authors declare that they have no competing interests.

## Authors’ contributions

KS, FK, IN and JN designed the study. KS implemented the software. FK developed the webpage. IN coordinated the study. KS, FK and IN wrote the manuscript. All authors read and approved the final manuscript.

## References

[B1] The New Science of Metagenomics: Revealing the Secrets of Our Microbial Planet 2007 Washington (DC) http://www.ncbi.nlm.nih.gov/books/NBK54006 21678629

[B2] Cox MP Peterson DA Biggs PJ SolexaQA: At-a-glance quality assessment of Illumina second-generation sequencing data BMC Bioinforma 2010 11 485 10.1186/1471-2105-11-485 PMC2956736 20875133

[B3] Schmieder R Edwards R Quality control and preprocessing of metagenomic datasets Bioinformatics 2011 27 6 863 864 10.1093/bioinformatics/btr026 21278185 PMC3051327

[B4] Altschul SF Gish W Miller W Myers EW Lipman DJ Basic local alignment search tool J Mol Biol 1990 215 3 403 410 2231712 10.1016/S0022-2836(05)80360-2

[B5] Eddy SR Accelerated Profile HMM Searches PLoS Comput Biol 2011 7 10 e1002195 10.1371/journal.pcbi.1002195 22039361 PMC3197634

[B6] Li H Homer N A survey of sequence alignment algorithms for next-generation sequencing Brief Bioinform 2010 11 5 473 483 10.1093/bib/bbq015 20460430 PMC2943993

[B7] Seshadri R Kravitz SA Smarr L Gilna P Frazier M CAMERA: a community resource for metagenomics PLoS Biol 2007 5 3 e75 10.1371/journal.pbio.0050075 17355175 PMC1821059

[B8] Markowitz VM Ivanova NN Szeto E Palaniappan K Chu K Dalevi D Chen IM Grechkin Y Dubchak I Anderson I IMG/M: a data management and analysis system for metagenomes Nucleic Acids Res 2008 36 D534 D538 Database issue 17932063 10.1093/nar/gkm869 PMC2238950

[B9] Meyer F Paarmann D D’Souza M Olson R Glass EM Kubal M Paczian T Rodriguez A Stevens R Wilke A The metagenomics RAST server - a public resource for the automatic phylogenetic and functional analysis of metagenomes BMC Bioinforma 2008 9 386 10.1186/1471-2105-9-386 PMC2563014 18803844

[B10] Yilmaz P Kottmann R Field D Knight R Cole JR Amaral-Zettler L Gilbert JA Karsch-Mizrachi I Johnston A Cochrane G Minimum information about a marker gene sequence (MIMARKS) and minimum information about any (x) sequence (MIxS) specifications Nat Biotechnol 2011 29 5 415 420 10.1038/nbt.1823 21552244 PMC3367316

[B11] Yilmaz P Gilbert JA Knight R Amaral-Zettler L Karsch-Mizrachi I Cochrane G Nakamura Y Sansone SA Glockner FO Field D The genomic standards consortium: bringing standards to life for microbial ecology ISME J 2011 5 10 1565 1567 10.1038/ismej.2011.39 21472015 PMC3176512

[B12] Huson DH Auch AF Qi J Schuster SC MEGAN analysis of metagenomic data Genome Res 2007 17 3 377 386 10.1101/gr.5969107 17255551 PMC1800929

[B13] Arumugam M Harrington ED Foerstner KU Raes J Bork P SmashCommunity: a metagenomic annotation and analysis tool Bioinformatics 2010 26 23 2977 2978 10.1093/bioinformatics/btq536 20959381

[B14] Parks DH Beiko RG Identifying biologically relevant differences between metagenomic communities Bioinformatics 2010 26 6 715 721 10.1093/bioinformatics/btq041 20130030

[B15] Kembel SW Cowan PD Helmus MR Cornwell WK Morlon H Ackerly DD Blomberg SP Webb CO Picante: R tools for integrating phylogenies and ecology Bioinformatics 2010 26 11 1463 1464 10.1093/bioinformatics/btq166 20395285

[B16] Dixon P VEGAN, a package of R functions for community ecology J Veg Sci 2003 14 6 927 930 10.1111/j.1654-1103.2003.tb02228.x

[B17] Caporaso JG Kuczynski J Stombaugh J Bittinger K Bushman FD Costello EK Fierer N Pena AG Goodrich JK Gordon JI QIIME allows analysis of high-throughput community sequencing data Nat Methods 2010 7 5 335 336 10.1038/nmeth.f.303 20383131 PMC3156573

[B18] Schloss PD Westcott SL Ryabin T Hall JR Hartmann M Hollister EB Lesniewski RA Oakley BB Parks DH Robinson CJ Introducing mothur: open-source, platform-independent, community-supported software for describing and comparing microbial communities Appl Environ Microbiol 2009 75 23 7537 7541 10.1128/AEM.01541-09 19801464 PMC2786419

[B19] Kanehisa M Goto S KEGG: Kyoto Encyclopedia of Genes and Genomes Nucleic Acids Res 2000 28 1 27 30 10.1093/nar/28.1.27 10592173 PMC102409

[B20] Tatusov RL Galperin MY Natale DA Koonin EV The COG database: a tool for genome-scale analysis of protein functions and evolution Nucleic Acids Res 2000 28 1 33 36 10.1093/nar/28.1.33 10592175 PMC102395

[B21] Finn RD Mistry J Tate J Coggill P Heger A Pollington JE Gavin OL Gunasekaran P Ceric G Forslund K The Pfam protein families database Nucleic Acids Res 2010 38 D211 D222 10.1093/nar/gkp985 19920124 PMC2808889

[B22] Haft DH Selengut JD White O The TIGRFAMs database of protein families Nucleic Acids Res 2003 31 1 371 373 10.1093/nar/gkg128 12520025 PMC165575

[B23] Qin J Li R Raes J Arumugam M Burgdorf KS Manichanh C Nielsen T Pons N Levenez F Yamada T A human gut microbial gene catalogue established by metagenomic sequencing Nature 2010 464 7285 59 65 10.1038/nature08821 20203603 PMC3779803

[B24] Ley RE Backhed F Turnbaugh P Lozupone CA Knight RD Gordon JI Obesity alters gut microbial ecology Proc Natl Acad Sci USA 2005 102 31 11070 11075 10.1073/pnas.0504978102 16033867 PMC1176910

[B25] Schwiertz A Taras D Schafer K Beijer S Bos NA Donus C Hardt PD Microbiota and SCFA in lean and overweight healthy subjects Obesity (Silver Spring) 2010 18 1 190 195 10.1038/oby.2009.167 19498350

[B26] Duncan SH Lobley GE Holtrop G Ince J Johnstone AM Louis P Flint HJ Human colonic microbiota associated with diet, obesity and weight loss Int J Obes (Lond) 2008 32 11 1720 1724 10.1038/ijo.2008.155 18779823

[B27] Sokol H Pigneur B Watterlot L Lakhdari O Bermudez-Humaran LG Gratadoux JJ Blugeon S Bridonneau C Furet JP Corthier G Faecalibacterium prausnitzii is an anti-inflammatory commensal bacterium identified by gut microbiota analysis of Crohn disease patients Proc Natl Acad Sci USA 2008 105 43 16731 16736 10.1073/pnas.0804812105 18936492 PMC2575488

